# Effects of different centrifugation conditions on clinical chemistry and Immunology test results

**DOI:** 10.1186/1472-6890-11-6

**Published:** 2011-05-10

**Authors:** Elisabeth I Minder, Adrian Schibli, Dagmar Mahrer, Predrag Nesic, Kathrin Plüer

**Affiliations:** 1 Stadtspital Triemli, Central laboratory Birmensdorferstrasse 497 CH-8063 Zürich Switzerland; 2 Stadtspital Triemli, Department for Internal Medicine Birmensdorferstrasse 497 CH-8063 Zürich Switzerland

## Abstract

**Background:**

The effect of centrifugation time of heparinized blood samples on clinical chemistry and immunology results has rarely been studied. WHO guideline proposed a 15 min centrifugation time without citing any scientific publications. The centrifugation time has a considerable impact on the turn-around-time.

**Methods:**

We investigated 74 parameters in samples from 44 patients on a Roche Cobas 6000 system, to see whether there was a statistical significant difference in the test results among specimens centrifuged at 2180 g for 15 min, at 2180 g for 10 min or at 1870 g for 7 min, respectively. Two tubes with different plasma separators (both Greiner Bio-One) were used for each centrifugation condition. Statistical comparisons were made by Deming fit.

**Results:**

Tubes with different separators showed identical results in all parameters. Likewise, excellent correlations were found among tubes to which different centrifugation conditions were applied. Fifty percent of the slopes lay between 0.99 and 1.01. Only 3.6 percent of the statistical tests results fell outside the significance level of p < 0.05, which was less than the expected 5%. This suggests that the outliers are the result of random variation and the large number of statistical tests performed. Further, we found that our data are sufficient not to miss a biased test (beta error) with a probability of 0.10 to 0.05 in most parameters.

**Conclusion:**

A centrifugation time of either 7 or 10 min provided identical test results compared to the time of 15 min as proposed by WHO under the conditions used in our study.

## 1. Background

Most clinical chemistry analyses in blood samples require centrifugation prior to the analyses in order to separate blood cells and other solid components such as fibrin from serum or plasma. Although this pre-analytical procedure is performed innumerable times every day in all medical laboratories worldwide, the influence of centrifugation on laboratory results has only rarely and only recently been investigated [1-3]. Indeed, the information contained in the publications and guidelines from scientific societies rely either on expert opinions or recommendations from the manufacturers rather than on published scientific investigations [4-6].

The separation efficacy of the centrifugation process depends on four variables, the centrifugation time, the relative centrifugation force (RCF), the length of blood collection tubes and the temperature. The length of tubes defines the volume of plasma or serum that is required for the analyses and is usually given. The RCF is limited by the resistance of the tubes and the blood components to the gravity and the technical limits of the centrifuge and its rotor. Variations in temperature are limited by stability of analytes. Thus, only the centrifugation time can be easily varied to achieve the desired quality of the specimen for the subsequent analyses.

The above cited guidelines proposed a centrifugation time of at least 10 min for serum and of 15 min for plasma with a RCF between 2000 and 3000 g [6]. Hospital laboratories serving emergency departments, intensive care units and busy outpatient clinics strive for minimizing their turn-around-times (TAT)[2]. The centrifugation step consumes a large portion of the pre-analytical time in the laboratory and therefore a considerable amount of the total TAT. A reduction in this time-consuming step is therefore in the focus. Indeed, centrifugation times as short as 30 sec have been used under certain circumstances, but no formal evaluations of such procedures have been published [7,8].

Another step to reduce TAT is the use of heparinized plasma instead of serum. Serum samples from fully anticoagulated patients may continue to coagulate for several hours after blood draw, which may affect the analytical process. Small particles of fibrin clots may block pipetting needles of the analyzers or interfere with chemiluminescence assays. Although collection tubes with special additives to reduce coagulation time to a few minutes, have been developed [3], yet we found that the coagulation process in specimens of fully anticoagulated patients is not completed after 15 min (E. Minder, unpublished observations). An alternative is to use tubes containing lithium heparinate which prevents coagulation and allows centrifugation immediately after the arrival of the tubes in the laboratory. Heparinized plasma instead of serum can be used for most clinical chemistry and many immunological analyses today, depending on the analytical platform and the reagents used.

Gels, also called separators, interpose between the cellular and fluid phase of the blood specimen during centrifugation and act as a barrier thereafter preventing the diffusion of analytes between the two phases. The gel barrier is formed only after adequate centrifugation time and force. An incomplete gel formation may interfere with certain analyses [9]. Separator gels from different manufacturers may have different compositions and therefore behave differently under identical centrifugation conditions, which could results in differences in test results.

In this report, we investigated the effect of distinct centrifugation conditions on a broad range of routine clinical chemistry and immunology analyses. These conditions included the centrifugation time pro-posed by the WHO-guidelines and two shorter ones of 10 and 7 min, respectively. We also examined the possible effect of two different gel separators on the same set of laboratory parameters.

## 2. Methods

### 2.1 Patients

Consecutive patients admitted to our medical wards between September and October 2009 were asked to provide six additional tubes for the study during regular blood draws. Exclusion criterion was a known or suspected anemia.

### 2.2 Blood sampling and centrifugation

Phlebotomy was performed using a butterfly needle (Greiner Bio-One reference number 450085). Between the regular drawing and the study samples, a 5 ml Z no additive Vacuette^® ^(Greiner Bio-One reference number 456202) was filled and discarded. Then, three evacuated tubes with gel separator (5 ml Lithium Heparin Sep 13 × 100 mm Vacuette^® ^Premium, Greiner Bio-One reference number 456083, available in Europe, called 'tube 11' hereafter) and three evacuated tubes with gel separator (5 ml Lithium Heparin Sep 13 × 100 mm Vacuette^®^, Greiner Bio-One reference number 456087 RP, available in USA, called 'tube 22' hereafter) were filled with venous blood. After gently inverting the tubes eight times, they were sent immediately to the laboratory by a pneumatic tube system and centrifuged within one hour after blood draw on a Rotixa 50 RS Hettich centrifuge equipped with a swing-out bucket rotor. The three centrifugation conditions were as follows: acceleration time of 32 sec (included in the overall centrifugation time), deceleration time of 32 sec (after centrifugation) and temperature at +15°C in all three conditions; condition 1: centrifugation time 15 min at 2180 g or 32,700 gmin, condition 2: 10 min at 2180 g or 21,800 gmin and condition 3: 7 min at 1870 g or 13,090 gmin, respectively.

The sequence of the tubes during blood draw was registered. To avoid any influence from this sequence, a pre-specified randomization protocol was used to allocate the tubes to the different centrifugation conditions. A collection of samples from at least 40 patients would attain sufficient statistical power and thus specimens from 47 patients were collected. Analyses were performed only if all six tubes collected per patient were of sufficient quality, i.e. without visible hemolysis or lipidemia. Tubes from 44 patients met the criteria and were therefore investigated. For statistical reasons, a patient was included only once.

### 2.3 Analyses of samples

Certified medical technicians with several years of working experience performed all laboratory procedures. Immediately after centrifugation, the samples were analyzed on a COBAS 6000 analyzer (Roche Diagnostics, Rotkreuz, Switzerland) equipped with the core unit cu 150 (Part number 727-0189), the modules c 501 (part number 727-2983) and e 601 (part number 727-2984). The instrument was equipped with standard reagents, calibrators and quality control material manufactured by Roche Diagnostics (Rotkreuz, Switzerland). Seventy-four different tests as listed in table 1 were performed on each tube, giving rise to a total of 444 results per patient. The analyses were performed within 3 hours after the sample collection with an exception of the immunological parameters for infectious diseases, which were examined within 48 hours. All tubes were stored at 4°C before analyses.

**Table 1:** All test analyzed, their control values and further data used for estimation of the beta error.

Test	Abbreviation	QC target value	unit	n	mean	SD	CV	Maximal error (CLIA)	lowest measured value	highest measured value	Range Ratio	estimated beta-error smaller than	Federal Register	Qualab	Clinical estimate
Albumin	Alb	47	g/L	16	47.44	**0.81**	1.71	3.65	25	48	1.92	<10%	**± 10%**	± 15%	
	
	Alb	30.9	g/L	18	31.71	**0.71**	2.28								

Alcaline Phosphatase	AP	71.9	U/L	18	70.56	1.04	**1.47**	67.80	36	416	11.56	<5%	**± 30%**	± 21%	
	
	AP	210	U/L	13	207.53	3.39	**1.63**								

Alanin aminotransferase	ALTL	45.5	U/L	17	45.65	0.49	**1.07**	36.10	7	354	50.57	<5%	**± 20%**	± 21%	
	
	ALTL	126	U/L	19	126.47	1.58	**1.25**								

Amikacin	Amik	7.83	umol/L	9	8.822	**1.057**	11.98	0.28	0	2.8	UD!	<10%			**10%**
	
	Amik	23.1	umol/L	9	23.828	**1.341**	5.63								
	
	Amik	46.3	umol/L	11	48.225	**2.994**	6.2								

Pancreatic Amylase	Amy-P	40	U/L	18	39.83	**0.71**	1.78	19.88	2	157	78.50	<5%		**± 25%**	
	
	Amy-P	107	U/L	19	107.11	**1.05**	0.98								

Amylase	Amyl	79.5	U/L	16	79.31	0.79	**1**	29.25	13	182	14.00	<5%	**± 30%**	± 30%	
	
	Amyl	198	U/L	12	195.56	1.42	**0.73**								

Aspartate aminotransferase	ASTL	47.3	U/L	17	46.59	1.18	**2.53**	25.40	10	244	24.40	<5%	**± 20%**	± 21%	
	
	ASTL	152	U/L	19	15.063	3.18	**2.11**								
	
	Bili-D	28.1	umol/L	19	28.326	1.125	3.97	22.20	1.1	220.9	200.82				**20%**

Total Bilirubin	Bili-T	16.5	umol/L	17	16.888	0.376	**2.23**	6.84	2.8	213.9	76.39	<5%	**± 6.84 umol/L or ± 20%**	± 20%	
	
	Bili-T	76.3	umol/L	19	75.421	5.257	**6.97**								

Calcium	Ca	2.13	mmol/L	18	2.1233	**0.0406**	1.91	0.25	1.87	2.71	1.45	<10%	± 0.25 umol/L	**± 12%**	
	
	Ca	3.41	mmol/L	21	3.3681	**0.0515**	1.53								

Carbamazepine	Carb	13.6	umol/L	9	11.6	**1.773**	15.28	0.13	0	1	UD	<5%	**± 25%**		
	
	Carb	37.9	umol/L	10	330.46	**3.45**	10.31								
	
	Carb	60.9	umol/L	11	54.955	**4.377**	7.96								

Cholinesterase	CHE	5400	U/L	16	5232.13	98.07	**1.87**	1216.80	2877	9291	3.23	<5%			**20%**
	
	CHE	1340	U/L	18	1313.78	26.47	**2.01**								

Cholesterol	Chol	2.65	mmol/L	17	2.718	**0.039**	1.43	0.51	2.3	7.8	3.39	<10%	**± 10%**	**± 10%**	

Cholesterol	Chol	5.27	mmol/L	18	5.117	**0.051**	1								

Creatinin Kinase	CK	152	U/L	18	148.78	1.7	**1.14**	69.60	12	452	37.67	<5%	**± 30%**	**± 30%**	
	
	CK	499	U/L	18	494.56	6.29	**1.27**								

CK, iso-enzyme MB	CKMB	5.13	ng/mL	9	5.43	0.063	**1.16**	1.45	0.9	21.2	23.56	?	**± 3 SD**		
	
	CKMB	56.7	ng/mL	7	55.62	0.903	**1.62**								

Bicarbonate	CO2	18.1	mmol/L	17	18.575	**0.331**	1.78	2.08	10.3	31.3	3.04	<10%			**10%**
	
	CO2	31.6	mmol/L	17	31.292	**0.63**	2.01								

Cortisol	Cort	300	nmol/L	16	312.6	**10.22**	3.27	190.83	110.6	1416	12.80	<5%	**± 25%**	± 20%	
	
	Cort	723	nmol/L	13	733.3	**12.22**	1.67								

Creatinine	Crea	89.1	umol/L	18	91.06	3.28	**3.6**	37.13	40	455	11.38	<5%	**± 26.5 umol/L or ± 15%**		
	
	Crea	346	umol/L	18	345.61	6.98	**2.02**								

C-reactive Protein	CRP	9.7	mg/L	15	9.653	0.223	**2.31**	16.26	0.3	216.5	721.67	<5%		**± 21%**	
	
	CRP	53.2	mg/L	16	52.706	2.088	**3.96**								

Chloride	Cl	84.8	mmol/L	28	83.709	**2.623**	3.13	5.15	87.9	118.1	1.34	>30%	**± 5%**	± 9%	
	
	Cl	118	mmol/L	28	114.967	**2.066**	1.8								

Digoxin	Digo	1.382/1.48	nmol/L	10		**0.048**	1.4	0.41	0.192	3.09	16.09	<5%	**± 25%**	± 24% (<1 nmol/L: ± 0.24%)	
	
	Digo	3.699	nmol/L	7	3.66	**0.084**	2.30								

Aethyl-ethanol	ETOH	11.3	mmol/L	15	11.253	0.767	**6.82**	0.16	-0.4	1.7	-4.25	<5%	**± 25%**		
	
	ETOH	33.2	mmol/L	14	33.35	2.69	**8.07**								

Ferritin	Ferri	12.5	ug/L	10	13.95	0.333	**2.39**	216.84	8.7	1726	198.39	<5%		**± 25%**	
	
	Ferri	416	ug/L	10	412.4	13.21	**3.2**								
	
	Ferri	1330	ug/L	12	1283	61.71	**4.81**								

Folic acid	Fol	6.765/7.08	nmol/L	11		0.639	**9.35**	5.48	9.44	45.4	4.81	<5%		**± 20%**	
	
	Fol	16.91	nmol/L	11	17.381	0.604	**3.48**								
	
	Fol	34.731	nmol/L	12	35.185	2.26	**6.42**								

Free Tri-iodo thyronine	FT3	6.1/6.33	pmol/L	17		0.157	**2.52**	0.84	1.63	6.78	4.16	<5%			**20%**
	
	FT3	25.6/26.0	pmol/L	15		0.6588	**2.658**								

Free Thyroxine	FT4	14.3	pmol/L	16	15.82	0.54	**3.41**	3.84	11.27	27.13	2.41	<5%	± 3 SD	**± 20%**	
	
	FT4	51.6	pmol/L	16	55.69	2.42	**4.35**								

Gentamycine	Gent	3.68	umol/L	9	3.189	**0.586**	18.38	1.33	0	10.6	UD	<5%	**± 25%**		
	
	Gent	9.38	umol/L	10	9.02	**0.944**	10.47								
	
	Gent	15.8	umol/L	11	15.891	**0.879**	5.53								

-glutamyl transpeptidase	GGT	46.4	U/L	17	45.65	0.61	**1.34**	195.30	9	1851	205.67	<5%		**± 21%**	
	
	GGT	206	U/L	18	205.89	2.85	**1.38**								

Glucose	Gluc	5.09	mmol/L	19	5.07	0.092	**1.81**	0.86	4.6	12.6	2.74	<10%	**± 0.333 mmol/L or ± 10%**	± 10%	
	
	Gluc	13.2	mmol/L	18	13.232	0.273	**2.06**								

Anti-Hepatitis A IgG	HAV	18.5/18.7/20.5	IU/L	13		0.505	**2.57**	6.30	3	60	20.00	<5%			**20%**
	
	HAV	32.1/33.5/37.1	IU/L	15		0.778	**2.39**								

Human chorionic gonadotropin, Subunit	HCG-beta	9.52	mIU/mL	17	9.278	0.21	**2.26**	2.90	0.1	23.1	231.00	<5%	± 3 SD	**± 25%**	
	
	HCG-beta	38.1	mIU/mL	16	36.654	1.018	**2.78**								

High-density lipoprotein	HDL	1.2	mmol/L	17	1.15	**0.0278**	2.42	0.41	0.12	2.63	21.92	<5%		**± 30%**	
	
	HDL	0.74	mmol/L	18	0.6461	**0.0154**	2.38								

Iron	Iron	20	umol/L	18	20.131	**0.304**	1.51	2.89	1.9	27	14.21	<5%	**± 20%**	**± 20%**	
	
	Iron	29.5	umol/L	19	29.684	**0.332**	1.12								

Potassium	K	3.41	mmol/L	27	3.3661	**0.08**	2.38	0.50	2.3	5.11	2.22	<10%	± 0.5 mmol/L	**± 9%**	
	
	K	6.26	mmol/L	28	6.1757	**0.0916**	1.48								

Lactate Dehydrogenase	LDH	318	U/L	16	321.38	3.74	**1.16**	143.10	232	1199	5.17	<5%	**± 20%**	± 21%	
	
	LDH	497	U/L	17	501.82	4.52	**0.9**								

Low-density lipoprotein	LDL	2.87	mmol/L	16	2.7144	**0.0669**	2.46	1.03	0.89	5.97	6.71	<5%			**30%**
	
	LDL	5.17	mmol/L	18	4.7817	**0.0932**	1.95								

Lipase	Lipe	55.4	U/L	18	54.28	0.67	**1.23**	97.68	6	808	134.67	<5%		**± 24%**	
	
	Lipe	89.9	U/L	18	88.33	1.88	**2.13**								

Magnesium	Mg	0.892	mmol/L	17	0.868	**0.0268**	3.09	0.26	0.51	1.56	3.06	<5%	**± 25%**	± 20%	
	
	Mg	1.68	mmol/L	17	1.7065	**0.0611**	3.58								

Myoglobin	Myo	85.2	ng/mL	11	80.107	2.025	**2.53**	28.68	21	170.2	8.10	<5%		**± 30%**	
	
	Myo	1080	ng/mL	11	1003.3	43.704	**4.36**								

Sodium	Na	121	mmol/L	27	119.79	**2.92**	2.44	12.69	129	153	1.19		± 4 mmol/L	**± 9%**	
	
	Na	145	mmol/L	28	142.69	**2.26**	1.58								

Pheno-barbital	Phno	39.9	umol/L	10	39.9	**1.91**	4.79	0.80	0	8	UD	<5%	**± 20%**		
	
	Phno	105	umol/L	11	97.55	**2.46**	2.52								
	
	Phno	215	umol/L	10	198.2	**5.2**	2.62								

Phosphate	Phos	1.29	mmol/L	17	1.3047	0.0194	**1.49**	0.20	0.62	2.06	3.32	<5%		**± 15%**	
	
	Phos	2.01	mmol/L	18	2.0472	0.0321	**1.57**								

N-terminal Brain natriuretic peptide	Pro-BNP	131	pg/mL	12	134	5.85	**4.37**	1837.05	5	24489	4897.80	<5%		**± 15%**	
	
	Pro-BNP	4360	pg/mL	11	4944	135.5	**2.74**								

Parathyroid-hormone	PTH	5.7982	pmol/L	7	5.47	0.165	**3.01**	5.13	2.12	40.61	19.16	<5%		**± 24%**	
	
	PTH	20.246	pmol/L	5	19.94	0.278	**1.4**								
	
	PTH	86.39	pmol/L	6	87.06	1.23	**1.42**								

Anti-Rubella IgG	RubG	3.57/3.96	IU/mL	13		0.186	**4.99**	50.02	0.17	500	2941.18		(1)		
	
	RubG	69.3	IU/mL	12	70.85	4.68	**6.61**								

Anti-Rubella IgM	RubM	0.23	COI	13	0.224	0.008	**3.57**				UD		(1)		
	
	RubM	2	COI	13	1.816	0.128	**7.05**								

Salicylate	Sali	0.29	mmol/L	9	0.2667	**0.0324**	12.15	-0.02	-0.22	0.05	-0.23	<5%			**20%**
	
	Sali	1.14	mmol/L	10	1.1335	**0.0362**	3.19								
	
	Sali	3.12	mmol/L	11	3.3218	**0.0795**	2.39								

Triiodo-thyronine	T3	2.46	nmol/L	16	2.34	0.179	**7.66**	0.30	0.541	2.47	4.57	<5%	± 3 SD		**20%**
	
	T3	5.59	nmol/L	16	5.48	0.383	**7**								

Theophyllin	Theo	30.1	umol/L	9	30.44	1.88	**6.18**	1.38	0	11	UD	<5%	**± 25%**		
	
	Theo	82.7	umol/L	10	83.1	2.51	**3.02**								
	
	Theo	171	umol/L	11	166.64	5.26	**3.16**								

Troponin T	TN-T	0.071	ug/L	13	0.073	0.003	**4.11**	0.31	0.01	2.54	254.00	<5%		**± 24%**	
	
	TN-T	2.24	ug/L	11	2.213	0.037	**1.67**								

High-sensitivity TNT	TNT-hs	0.0302	ug/L	11	0.032	0.001	**3.13**	0.26	0.003	2.19	730.00	<5%		**± 24%**	
	
	TNT-hs	2.45	ug/L	11	2.488	0.054	**2.17**								

Tobramycin	Tobr	1.79	ug/mL	9	1.8	0.071	**3.94**	0.03	0	0.2	UD	<5%	**± 25%**		
	
	Tobr	4.01	ug/mL	10	3.94	0.07	**1.78**								
	
	Tobr	8.17	ug/mL	11	7.9	0.261	**3.30**								

Anti-Toxo-plasma IgG	ToxoG	0.84	IU/mL	13	0.899	0.051	**5.67**	77.25	0.13	617.9	4753.08	<5%			**20%**
	
	ToxoG	48.1	IU/mL	13	50.549	2.93	**5.80**								

Anti-Toxo-plasma IgM	ToxoM	0.18	COI	13	0.14	**0.015**	10.71		-1	-1	1.00				
	
	ToxoM	1.78	COI	13	1.86	**0.057**	3.06								

Total Protein	TP	64.6	g/L	14	65.786	1.122	**1.71**	7.00	50	90	1.80	<10%	**± 10%**	± 15%	
	
	TP	102	g/L	15	105.23	1.05	**1.00**								
	
	TP	66.8	g/L	17	66.88	0.86	**1.29**								
	
	TP	50.1	g/L	19	50.11	0.74	**1.48**								

Triglycerides	Trigl	1.22	mmol/L	17	1.2371	0.0126	**1.02**	0.74	0.67	5.21	7.78	<5%	**± 25%**	± 20%	
	
	Trigl	2.37	mmol/L	18	2.3711	0.0345	**1.46**								

Transferrin	Trfe	33	umol/L	13	33.154	**0.689**	2.08	6.30	16	47	2.94	<5%			**20%**
	
	Trfe	48.9	umol/L	15	48.23	**0.68**	1.41								
	
	Trfe	25.1	umol/L	17	25.41	**0.51**	2.01								

Thyroid-stimulating hormone	TSH	1.65	uIU/mL	16	1.57	0.019	**1.21**	0.62	0.05	6.14	122.80	<5%	± 3 SD	**± 20%**	
	
	TSH	9.09	uIU/mL	15	8.57	0.114	**1.33**								

Uric Acid	UA	275	umol/L	17	276.12	2.71	**0.98**	75.00	93	907	9.75	<5%	± 17%	**± 15%**	
	
	UA	579	umol/L	18	575.11	10.53	**1.83**								

Urea	Ureal	6.84	mmol/L	17	6.9	0.094	**1.36**	4.15	2	39.5	19.75	<5%		**± 20%**	
	
	Ureal	24.4	mmol/L	18	23.967	0.387	**1.61**								

Valproate	Valp	238	umol/L	9	233.44	**29.75**	12.74	3.88	0	31	UD	<5%	**± 25%**		
	
	Valp	519	umol/L	10	549	**47.71**	8.69								
	
	Valp	811	umol/L	11	857.45	**69.14**	8.06								

Vanco-mycin	Vanc	4.22	umol/L	10	4.59	**0.468**	10.2	0.18	0	1.8	UD	<5%			**20%**
	
	Vanc	13.5	umol/L	10	15.52	**0.914**	5.89								
	
	Vanc	19.6	umol/L	12	22.083	**0.997**	4.51								

Vitamin B12	VitB12	177.858/194.8	pmol/L	11		**9.546**	5.24	123.35	109.5	1124	10.26	<5%		**± 20%**	
	
	VitB12	392.616/408.9	pmol/L	11		**11.8**	2.99								
	
	VitB12	856.08	pmol/L	12	859.636	**20.04**	2.33								

Vitamin D	VitD	55	nmol/L	6	48.36	6.93	**14.33**	14.71	17.78	129.3	7.27	<5%		**± 20%**	
	
	VitD	83.75	nmol/L	4	72.95	6.92	**9.49**								
	
	VitD	115.5	nmol/L	6	106.5	10.31	**9.68**								

(1) Target value ± 2 dilutions or immune or nonimmune or positive or negative, UD = undetermined

All analyses were listed including their abbreviations used in the further tables. Their standard deviations (SD) and coefficient of variations (CV) of the controls (QC) are given. The target values of the QC material of different analytes, as well as the measurement units, the number of QC repetitions (n), the mean, SD and CV of the QC materials are displayed. The beta error was estimated [12] using the range ratio and the allowable biases as defined by the federal registry, the Qualab or the clinical estimate. The values used for the evaluations are displayed in bold.

This study was initiated by the investigators; the costs of the study were covered by two companies (see acknowledgments). The study was approved by the institutional review board (Kantonale Ethikkommission Zürich), and the participating patients gave their written informed consent. Their samples were immediately anonymized after blood draw, but were decoded upon request of the patients.

### 2.4 Statistics

Analyse-it for Excel (Version 2.11, 2008) was used for the statistical analyses; the statistic procedure applied being the Deming fit. In contrast to linear regression analysis, the Deming procedure allows for a random error in both test and reference measurements [10]. Performed with weighed function, it accounts also for a non-constant random error over the measurement range [11,12]. A two sided p value of < 0.05 was considered as significant. Coefficients of variation (CV) and standard deviations (SD) were calculated by the Cobas 6000 software from the quality control samples as mentioned in table 1. These quality controls were run once a day.

#### 2.4.1 Determination of the alpha error

The triplicates of each parameter of tubes 11 (comprising all different centrifugation conditions) per patient were compared with those of tube 22, to detect variations caused by the different gel separators. Second, test results from all centrifugation conditions within one tube were compared among each other. As singletons were compared, SD or CV had to be defined using the values of the quality control samples displayed in table 1. If the SD's were approximately constant over the measured range, the mean of SD's of those quality control samples were entered. In case CV was more constant than SD, a weighed Deming fit was applied using the means of CV's of those quality control samples.

#### 2.4.2 Determination of the beta error

The null hypothesis of the Deming fit tests the identity of a test method to a reference method. The p value defines the probability (alpha error) that the two methods deliver identical results. However, it does not determine the probability to detect a deviation of the test method from the reference method when in fact the test method produces aberrant or biased results. The probability to miss an existent bias (beta error) depends on the measurement range, the allowable bias, the analytical measurement error and the distribution of the data [12]. First, we estimated the beta error based on the tables five and six of publication [12], using the measurement range of our data. As allowable bias the values were primarily taken from Federal registry [13], secondarily from the Swiss regulation of Qualab [14], and thirdly by an estimate of clinical requirement by one of the investigators (EIM) The data used are highlighted on table 1. Second, we calculated the deviation or bias of the test method to the reference method for the limits of reference values. The calculated bias was compared to an allowable bias of 5%, 10% 20%, 30% or 40% and the probability was determined that the calculated bias exceeds the allowable bias thus indicating a significant deviation of the test method from the reference method.

## 3. Results

**Figure 1:**
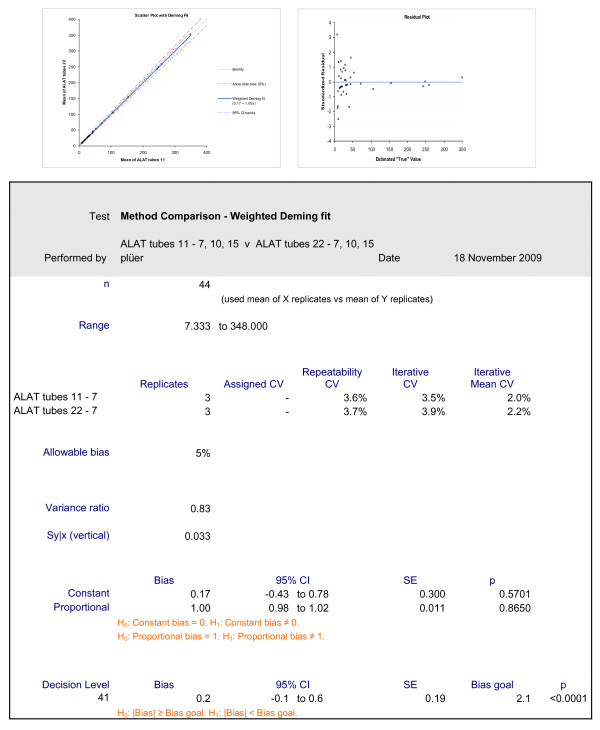
**Deming fit**. This figure shows a typical report of the Deming fit by Analyse-it for Excel: Graph A represents the scatter plot, the regression line and indicates the identity line and the limits of a 5% bias. Graph B plots the standardized residuals. The statistical information as discussed in the text is given as a table.

As an illustrative example of the applied Deming method, the scatter plot, the residual plot and the correspondent numeric output of alanin-amino-transferase (ALAT) measurements are shown in Figure 1. The means of the triplicates of tube 11 are compared with those of tube 22. Figure 1A shows that the identity and the weighted Deming regression line lie closely to each other. Figure 1B illustrates that the deviation of the standardized residuals strongly increase in lower values, indicating that a weighted rather than unweighted procedure should be applied. On the lower part of Figure 1, the numerical results of the constant and proportional bias and their p values are displayed. The constant bias corresponding to the intercept in regression analysis should be 0.00 ideally, the proportional bias corresponding to the slope 1.00. The p value of 0.57 for the constant bias and that of 0.87 for the proportional bias indicate that neither the slope nor the intercept differs significantly from their ideal values.

A compilation of the constant and proportional bias over all statistical tests including all parameters and all centrifugation conditions is displayed in additional file 1, table S1 and an excerpt of them is illustrated in table 2. The leftmost column shows the parameter analyzed. For each parameter, seven statistical comparisons as displayed in the adjacent columns were made, resulting in 357 Deming tests (51 parameters × 7 comparisons). One tube 22, condition 2 was excluded because several analytical parameters showed aberrations that exceeded 3 standardized residuals, and we concluded that this tube did not contain a sample of appropriate quality. Twenty-three analytical parameters could not be evaluated, as either, they were not quantitative tests or most patient samples did not contain measurable amounts and therefore a reliable quantitative comparison could not be performed. They are listed as footnotes in additional file 1, table S1.

**Table 2:** An illustrative excerpt of all Deming fits containing the data of some clinical chemistry and immunology analytes.

Analysis	11:22		11 - 15:10		11 - 15:7		11 - 10:7		22 - 15:10		22 - 15:7		22 - 10:7	
	**Bias** **Const**.	**Prop**.	**Bias** **Const**.	**Prop**.	**Bias** **Const**.	**Prop**.	**Bias** **Const**.	**Prop**.	**Bias** **Const**.	**Prop**.	**Bias** **Const**.	**Prop**.	**Bias** **Const**.	**Prop**.

	**95% CI**	**95% CI**	**95% CI**	**95% CI**	**95% CI**	**95% CI**	**95% CI**	**95% CI**	**95% CI**	**95% CI**	**95% CI**	**95% CI**	**95% CI**	**95% CI**

Alb	-1.81	1.06	-1.86	1.06	-1.05	1.03	0.76	0.98	-0.16	1.01	0.25	0.99	0.40	0.99

Alb	-4.33 to 0.71	0.99 to 1.13	-4.52 to 0.79	0.98 to 1.13	-2.55 to 0.45	0.99 to 1.07	-1.90 to 3.41	0.90 to 1.05	-2.54 to 2.23	0.94 to 1.07	-1.85 to 2.34	0.94 to 1.05	-1.83 to 2.63	0.93 to 1.04

Bili-T	-0.08	1.01	-0.15	1.03	0.10	1.00	0.24	0.96	-0.04	1.01	-0.11	1.03	-0.06	1.02

Bili-T	-0.18 to 0.01	0.99 to 1.02	-0.44 to 0.15	1.00 to 1.06	-0.18 to 0.38	0.96 to 1.03	0.04 to 0.44	0.94 to 0.99	-0.39 to 0.31	0.97 to 1.05	-0.52 to 0.29	0.99 to 1.07	-0.45 to 0.32	0.98 to 1.07

Ca	-0.05	1.03	-0.17	1.08	-0.18	1.08	-0.01	1.00	-0.09	1.04	-0.05	1.02	0.02	0.99

Ca	-0.15 to 0.04	0.99 to 1.07	-0.35 to 0.02	0.99 to 1.16	-0.33 to -0.02	1.01 to 1.15	-0.13 to 0.11	0.95 to 1.05	-0.24 to 0.05	0.98 to 1.10	-0.20 to 0.09	0.96 to 1.09	-0.12 to 0.16	0.93 to 1.05

CK	0.15	1.00	-0.40	1.01	-1.63	1.02	-1.20	1.01	-0.98	1.03	-0.66	1.02	0.31	0.99

CK	-0.19 to 0.49	0.99 to 1.01	-1.43 to 0.62	1.00 to 1.03	-2.54 to -0.71	1.00 to 1.03	-2.89 to 0.50	0.98 to 1.03	-2.80 to 0.85	1.00 to 1.06	-1.48 to 0.17	1.00 to 1.03	-2.11 to 2.74	0.95 to 1.03

CKMB	0.23	0.94	-0.62	1.15	-0.15	1.01	0.42	0.87	-0.11	1.03	-0.10	1.01	0.01	0.98

CKMB	-0.11 to 0.58	0.85 to 1.02	-2.29 to 1.05	0.74 to 1.56	-0.40 to 0.09	0.96 to 1.06	-1.06 to 1.89	0.50 to 1.24	-0.55 to 0.33	0.91 to 1.14	-0.64 to 0.44	0.87 to 1.15	-0.26 to 0.29	0.92 to 1.05

FT3	0.08	0.98	-0.18	1.05	-0.20	1.06	-0.01	1.00	-0.08	1.05	-0.24	1.07	-0.06	1.02

FT3	-0.10 to 0.26	0.93 to 1.03	-0.36 to -0.00	1.00 to 1.11	-0.42 to 0.03	0.99 to 1.12	-0.27 to 0.24	0.93 to 1.08	-0.52 to 0.17	0.96 to 1.14	-0.67 to 0.20	0.95 to 1.18	-0.28 to 0.17	0.96 to 1.08

FT4	-0.22	1.02	-0.69	1.05	-0.76	1.05	-0.05	1.00	-0.56	1.04	0.73	1.04	-0.17	1.01

FT4	-0.63 to 0.19	0.99 to 1.04	-1.88 to 0.51	0.97 to 1.12	-2.40 to 0.89	0.95 to 1.15	-1.60 to 1.49	0.90 to 1.09	-1.45 to 0.34	0.98 to 1.09	-2.01 to 0.54	0.97 to 1.12	-1.07 to 0.74	0.95 to 1.06

GGT	0.47	1.00	-0.23	1.01	0.22	1.00	0.44	0.99	-0.15	1.01	-0.95	1.02	-0.78	1.01

GGT	0.12 to 0.83	0.98 to 1.01	-1.26 to 0.80	0.99 to 1.03	-0.22 to 0.65	0.99 to 1.02	-0.35 to 1.22	0.97 to 1.01	-1.00 to 0.70	1.00 to 1.03	-2.37 to 0.47	1.00 to 1.05	-2.53 to 0.96	0.98 to 1.04

HCGbeta	0.00	1.01	-0.01	1.01	0.00	0.98	0.00	0.97	0.00	1.01	0.00	1.02	0.00	1.01

HCGbeta	-0.01 to -0.00	0.98 to 1.03	-0.01 to 0.00	0.97 to 1.05	-0.01 to 0.00	0.94 to 1.02	0.00 to 0.01	0.92 to 1.02	-0.01 to 0.00	0.97 to 1.04	-0.01 to 0.00	0.98 to 1.05	0.00 to 0.00	0.97 to 1.05

In the second column from the left (headed 11:22), the triplicates of tubes '11' are compared with the triplicates of tube 22. The constant bias (i.e. intercept) and its 95% confidence intervals (CI) as well as the proportional bias (i.e. slope) and its confidence interval are listed. The confidence intervals for the constant bias should include 0.00, and that of proportional bias 1.00, in order to prove the identity of the methods. The columns to the right contain identical data for comparison, 15 versus 10 min centrifugation, 15 versus 7 and 10 versus 7 min. Further to the right, the same comparisons were made for the '22'-tubes. The full list of comparisons is given as table S1 in additional files.

Table 2 and additional file 1, table S1 list the statistical evaluations on all investigated parameters including the constant bias, the proportional bias and their 95% confidence intervals. The 95% confidence intervals included the ideal values (i.e. 0.00 in constant bias or 1.00 in proportional bias) for 688 of the 714 results (357 each constant and proportional bias), and not included in this interval were 26 results (18 constant, 8 proportional biases). These data correspond to 3.6% of the 714 results, 5.0% of constant and 2.2% of proportional biases, respectively. These percentages do not surpass the expected 5%, as the confidence intervals include only 95% and not 100% of random variations. Moreover, aberrations from the ideal values were minor in these cases, supporting the concept of pure randomness.

**Figure 2:**
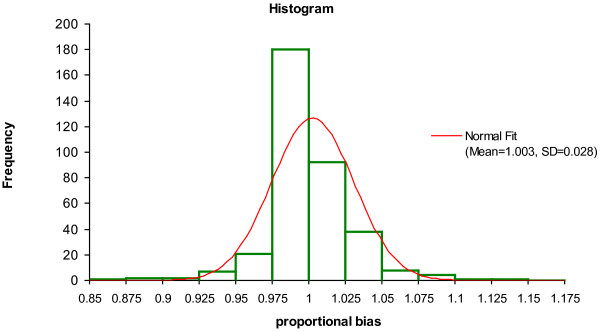
**Distribution of proportional bias: Histogram showing the distribution of the proportional biases (Slopes)**. The slopes scatter around the ideal value of 1.00. For comparison, a normal distribution is depicted.

To further analyze the results, the distribution of the proportional biases that should randomly vary around the value 1.00 was studied (Figure 2). This analysis showed that 50 percent of all proportional bias (slopes) were located between 0.990 and 1.010, and 99% of them were located between 0.924 and 1.086. The extreme values were 0.90 and 1.15. The parameters with proportional biases outside the 99% distribution and below 0.924 were the following: sodium, bicarbonate, CKMB, and those above 1.086 were bicarbonate and CKMB. Apparently, the Deming fit did not appropriately estimate the slope in some of these outliers such as CKMB. From the scatter plot, we assume that relatively large variations in low normal values influenced this estimate. In others, like bicarbonate or sodium, the analytical imprecision combined with a small measurement range resulted in aberration from the 95% confidence interval.

As the values of the constant biases (intercepts) depend on the measurement range of the analyte, no similar analysis could be performed. Instead, we analyzed whether deviations accumulate in a certain centrifugation condition. The 95% confidence interval of the constant bias did not include the ideal 0.00 value 18 times, namely in comparison between tube 11 and tube 22: twice; in tube 11 - between centrifugation condition 1 and 2: once; between condition 1 and 3: 3 times; between condition 2 and 3: 3 times; in tube 22 - between centrifugation condition 1 and 2: 4 times; between condition 1 and 3: twice and between condition 2 and 3: 3 times. Thus, the aberrant values did not cluster under any centrifugation condition.

**Figure 3:**
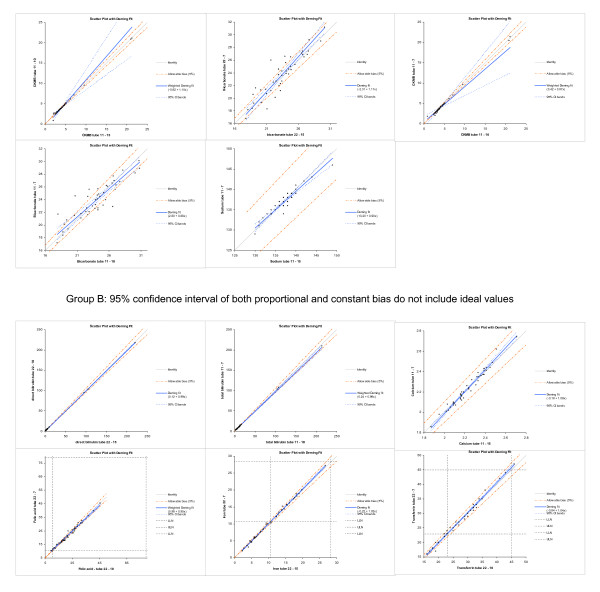
**The scatter plots of the Deming fits mostly aberrant from identity are depicted**. The upper five diagrams (group A) show those tests with a proportional bias outside the 99% distribution range (s. figure 2). The lower six diagrams (group B) show tests of which both proportional and constant bias did not indicate identity between the test and the reference method, as listed in table 3.

In six instances, both constant and proportional bias confidence intervals did not include the ideal value. These tests were therefore considered as potentially significantly aberrant. These conditions were listed in table 3. In all but one case, condition 3 was involved as test method, whereby either condition 1 or 2 were reference. As in all instances, the confidence intervals of the proportional bias and constant bias did not include the ideal values only marginally, we concluded that these outliers were generated only by chance. In order to enable the readers to make his or her own adjudgment on the significance of these deviations, the scatter plots of all six conditions including the slopes, their confidence intervals, the identity lines and the upper and lower limits of reference where appropriate, are displayed in Figure 3.

**Table 3:** Tests with confidence intervals of both proportional and constant bias exceeding the confidence interval and indicating a possible lack of identity between test and reference method.

Parameter	Condition	Constant bias	Proportional bias
Bilirubin direct	T 22; C 1 : C 2	0.12 (0.06 to 0.18)	0.99 (0.98 to 0.99)

Bilirubin total	T 11; C 2 : C 3	0.24 (0.04 to 0.44)	0.96 (0.94 to 0.99)

Calcium	T 11; C 1 : C 3	-0.18 (-0.33 to -0.02)	1.08 (1.01 to 1.15)

Folic acid	T 22; C 2 : C 3	0.98 (0.24 to 1.72)	0.95 (0.91 to 0.99)

Iron	T 22; C 1 : C 3	-0.22 (-0.43 to -0.02)	1.02 (1.01 to 1.03)

Transferrin	T 22; C 2 : C 3	-0.84 (-1.67 to -0.02)	1.04 (1.01 to 1.06)

T: tube; C: centrifugation condition as listed under section 2: Patients, materials and methods.

The procedure that has been discussed so far, calculates the probability of identity between the test method and the reference method (alpha error). For the purpose of this study however, the probability of a deviation between the two methods i.e. the beta error, is at least as relevant as the alpha error. The use of patient samples with a sufficiently large measurement range thereby enforced the statistical power.

The first estimate of the beta error according to the description in paragraph 2.4.2 is listed per parameter in table 1. All parameters except for chloride were below the allowable limits of the bias, the problem in chloride being the physiologically narrow range of sample values. Second, it was tested whether a specified bias could be detected at the limits of the reference values. As illustrated in Figure 1, a bias, named allowable bias on the figure, was pre-specified and the probability calculated that such a bias could be detected. In this example, the upper limit of reference is 41 U/L. Five percent of 41 are 2.1 indicated on the figure as bias goal. The bias calculated from the data corresponds to 0.2, which is much smaller than 2.1. The null hypothesis that the bias is equal or larger than the bias goal can be falsified with high probability, which excludes a deviation with high probability.

An illustrative sample of the 539 reference limits tested is given in table 4 and the full information is listed in additional file 2, table S2. 88.9% or 479 of those tests would detect a 5% bias. Interestingly, chloride was within this group. If the allowable bias was set to 10%, 20%, 30% and 40%, such biases would be excluded at further 48, 8, 3, and 1 reference limits, respectively. These figures correspond to a cumulative frequency of 97.8%, 99.2%, 99.8% and 100% of all levels tested. The cortisol test required the highest allowable biases of all tests for falsifying the null hypothesis, namely, once 40%, three times 30%, twice 20% and once 10%. This indicates that the number of specimens tested were insufficient for cortisol to exclude a bias with sufficient certainty.

**Table 4:** Excerpt of a list of the probabilities to detect a 5% bias with 95% certainty at the limits of the reference ranges limited to the comparison of tubes 11 to tubes 22.

	11:22							11:22					
Analyse	Ref. limits	Bias	95% Cl	SE	Bias Goal	p	Analyse	Ref. limits	Bias	95% Cl	SE	Bias Goal	p
Alb	35	0.3	0.0 to 0.5	0.12	1.8	<0.0001	HDL	0.9	0.0	0.0 to 0.0	0.00	0.0	<0.0001
Alb	58	1.6	-0.1 to 3.3	0.85	2.9	0.0683	Iron	10.6	0.1	0.0 to 0.2	0.05	0.5	<0.0001
AP	117	0.5	-0.3 to 1.2	0.39	5.9	<0.0001	Iron	28.3	0.4	0.1 to 0.8	0.17	1.4	<0.0001
ALTL	41	0.2	-0.1 to 0.6	0.19	2.1	<0.0001	K	3.5	0.0	0.0 to 0.1	0.01	0.2	<0.0001
Amy-P	53	0.0	-0.4 to 0.5	0.23	2.7	<0.0001	K	4.5	0.0	0.0 to 0.1	0.03	0.2	<0.0001
Amyl	100	1.0	0.3 to 1.7	0.35	5.0	<0.0001	LDH	288	10.1	0.9 to 19.3	4.56	14.4	0.1746
ASTL	37	0.2	-0.2 to 0.6	0.19	1.9	<0.0001	LDL	3.9	0.1	0.0 to 0.2	0.04	0.2	0.0019
Bili-D	2	0.0	0.0 to 0.1	0.03	0.1	0.0039	Lipe	60	0.5	0.0 to 1.1	0.28	3.0	<0.0001
Bili-T	20	0.0	-0.2 to 0.3	0.13	1.0	<0.0001	Mg	0.75	0.0	0.0 to 0.0	0.00	0.0	0.0138
Ca	2.02	0.0	0.0 to 0.0	0.01	0.1	<0.0001	Mg	1.25	0.0	0.0 to 0.0	0.01	0.1	<0.0001
Ca	2.6	0.0	0.0 to 0.0	0.01	0.1	<0.0001	Myo	28	0.2	-0.2 to 0.5	0.16	1.4	<0.0001
CHE	3000	-7.7	-61.1 to 45.7	26.47	150.0	<0.0001	Myo	72	-0.3	-0.9 to 0.3	0.31	3.6	<0.0001
CHE	11000	132.0	8.9 to 255.0	60.99	550.0	<0.0001	Na	135	0.1	-0.2 to 0.3	0.13	6.8	<0.0001
Chol	3.1	0.0	-0.1 to 0.0	0.03	0.2	<0.0001	Na	145	-0.5	-1.2 to 0.2	0.34	7.3	<0.0001
Chol	6.5	0.1	0.0 to 0.2	0.07	0.3	0.0011	Phos	0.87	0.0	0.0 to 0.0	0.01	0.0	<0.0001
CK	195	0.8	-0.9 to 2.5	0.82	9.8	<0.0001	Phos	1.45	0.0	0.0 to 0.0	0.01	0.1	<0.0001
CKMB	4.94	-0.1	-0.2 to 0.0	0.04	0.2	<0.0001	Pro-BNP	125	0.3	-0.9 to 1.6	0.63	6.3	<0.0001
CO2	22	-0.1	-0.4 to 0.1	0.11	1.1	<0.0001	PTH	1.59	0.0	-0.1 to 0.1	0.04	0.1	0.0435
CO2	29	-0.4	-0.7 to -0.0	0.17	1.5	<0.0001	PTH	9.33	-0.1	-0.3 to -0.0	0.05	0.5	<0.0001
Cort	82	0.9	-5.3 to 7.1	3.07	4.1	0.1509	RubG	10	0.0	-0.4 to 0.4	0.19	0.5	0.0111
Cort	958	0.3	-10.4 to 10.9	5.27	47.9	<0.0001	T3	1.3	0.0	0.0 to 0.0	0.01	0.1	<0.0001
Crea	59	0.1	-0.4 to 0.7	0.29	3.0	<0.0001	T3	3.1	0.0	-0.1 to 0.1	0.05	0.2	0.0054
Crea	104	0.0	-0.5 to 0.4	0.23	5.2	<0.0001	TN-T	0.01	0.0	0.0 to 0.0	0.00	0.0	<0.0001
CRP	10	0.1	-0.1 to 0.2	0.08	0.5	<0.0001	TNT-hs	0.014	0.0	0.0 to -0.0	0.00	0.0	<0.0001
Cl	97	0.1	-0.3 to 0.5	0.18	4.9	<0.0001	ToxoG	1	0.0	0.0 to 0.0	0.00	0.1	<0.0001
Cl	110	0.0	-0.4 to 0.4	0.20	5.5	<0.0001	TP	66	0.4	0.0 to 0.8	0.19	3.3	<0.0001
Ferri	30	-0.1	-0.4 to 0.2	0.15	1.5	<0.0001	TP	87	0.8	-0.4 to 2.0	0.59	4.4	<0.0001
Ferri	400	2.7	-0.9 to 6.2	1.77	20.0	<0.0001	Trigl	2.3	0.0	0.0 to 0.0	0.01	0.1	<0.0001
Fol	10.4	-0.1	-0.6 to 0.4	0.24	0.5	0.0484	Trfe	23	0.1	-0.1 to 0.3	0.09	1.2	<0.0001
Fol	78.9	1.6	-0.9 to 4.1	1.25	3.9	0.0343	Trfe	45	1.0	-0.2 to 2.3	0.62	2.3	0.0273
FT3	3.1	0.0	0.0 to 0.1	0.02	0.2	<0.0001	TSH	0.34	0.0	0.0 to 0.0	0.01	0.0	0.0873
FT3	6.8	-0.1	-0.2 to 0.1	0.07	0.3	0.0003	TSH	5.6	0.0	-0.1 to 0.1	0.05	0.3	<0.0001
FT4	12	0.0	-0.1 to 0.1	0.06	0.6	<0.0001	UA	420	0.9	-0.4 to 2.2	0.63	21.0	<0.0001
FT4	22	0.2	-0.1 to 0.4	0.11	1.1	<0.0001	Ureal	3	0.0	0.0 to 0.0	0.01	0.2	<0.0001
GGT	49	0.2	-0.1 to 0.6	0.18	2.5	<0.0001	Ureal	8	0.0	0.0 to 0.0	0.01	0.4	<0.0001
Gluc	3.9	0.0	-0.1 to 0.1	0.06	0.2	0.0029	VitB12	133	1.8	-2.5 to 6.1	2.15	6.7	0.0144
Gluc	5.8	0.0	0.0 to 0.1	0.04	0.3	<0.0001	VitB12	675	0.8	-7.7 to 9.3	4.20	33.8	<0.0001
HAV	20	0.0	-0.1 to 0.1	0.04	1.0	<0.0001	VitD	75	0.6	-1.0 to 2.3	0.80	3.8	0.0002
HCGbeta	2	0.0	0.0 to 0.1	0.02	0.1	0.0002							

Those conditions not sensitive enough to detect a 5% bias were retested for a 10, 20, 30 or 40% bias (data not shown). The column heading Ref. limits means limits of reference ranges. At these concentrations of the analyte, the probability was tested to detect a 5% bias. Bias: The calculated deviation of the Deming regression line and its 95% confidence intervals (CI) and the standard error are given. Bias goal corresponds to a 5% deviation of the Deming regression line from identity at the tested analyte concentration. The full information on all comparisons is given in supplemental Table S2.

We conclude that the conditions used including the number of measurements, the analytical range and the analytical imprecision were sufficient to detect a beta error with sufficient probability.

## 4. Discussion

Our study shows that the three centrifugation conditions tested deliver identical results. Moreover, the statistical power was sufficient to exclude any major deviation with a high probability. As mentioned before, very few studies on the influence of different centrifugation conditions on laboratory test results have so far been published [1-3]. In addition, a number of unpublished investigations were made available to us by the manufacturers of tubes or reagents. Our study results do not contradict any of those studies, but rather, they extent both the number of parameters and the test ranges. The number of samples and the spectrum of tests were relatively small in all preceding studies and, as the samples mostly from healthy persons were analyzed, the measurement ranges were relatively narrow, which altogether resulted in a relatively large beta-error. However, no beta error was ever calculated in the previous studies despite its significance.

In this study, a total of 14690 data pairs of quantitative clinical chemistry and immunology tests, acquired by comparing six different conditions and three different centrifugation regimens, were analyzed in 357 Deming procedures. Most of the comparisons showed excellent reproducibility indicating that the different centrifugation conditions used did not affect the outcome of laboratory testing. The percentage of statistical tests outside the 95% confidence limits was below the number expected for random variations. Corrections of the p-values for multiple testing could be performed, such as the Bonferroni corrections. However, such procedure would increase the beta-error that, in our opinion, is at least as informative as the alpha-error for the purpose of our study. We preferred to analyze those outlier data to see whether they show any evidence of significant bias, and found evidence of random aberration rather than any statistical significant bias. The only way to ascertain our conclusion on the randomness of outliers would be a repetitious examination under identical conditions to exclude or confirm those aberrant values. We believe that we have provided sufficient data to convince the readers on randomness of these outliers and that the three centrifugation conditions as well as the two different gel separators of Greiner Bio-One provided identical results on a Cobas 6000 system of Roche Diagnostics.

The beta errors calculated by two separate methods confirmed a sufficient power of our analyses to detect significant deviations. The first of the two methods relied on regulatory limits either from USA or from Switzerland supplemented with our clinical estimates. The second one tests the biases at the limits of reference values to see whether they exceed a pre-specified bias. The reference limits were chosen for this purpose, as they discriminate between "normal" and "pathological" values, and deviations at these limits would result in "falsely normal" or "falsely pathological" results.

In addition to the quantitative tests, data from 23 qualitative tests or quantitative tests with little or no measurable concentrations did not provide any discrepant results. However, these tests were not statistically evaluated.

## 5. Conclusions

Our study provided substantial evidences that the centrifugation condition from the WHO guideline, the conditions of 10 min centrifugation time at 2180 g and of 7 min at 1870 g were equally effective to the performance of the subsequent laboratory analyses. Each of these conditions can be applied to a broad range of clinical chemistry and immunology tests, provided that specified tubes and analytical conditions were used (see section 2). Laboratories that have been hitherto accommodating the WHO recommendations are now having the possibility of reducing their centrifugation time to less than half of the original amount and in turn, to reduce their overall TAT significantly. Those laboratories that so far are reluctant to switch from serum to heparinized plasma because of the prolonged centrifugation time, can now have a second thought.

## Competing interests

The authors declare that they have no competing interests.

## Authors' contributions

EIM designed the study and wrote the manuscript, AS organized and supervised the blood drawings on the wards, DM and PN participated in the design of the study, organized and supervised the sample centrifugation and analyses of the samples, KP collected the data and performed the statistical analyses. All authors read and approved the final manuscript.

## Pre-publication history

The pre-publication history for this paper can be accessed here:

http://www.biomedcentral.com/1472-6890/11/6/prepub

## Supplementary Material

Additional file 1 **(Table S1): Deming fit results from all analytical parameters and centrifugation conditions**. The results are displayed as outlined in the legend to table 2. Click here for file

Additional file 2 **(Table S2): Probabilities to detect a 5% bias with 95% certainty at the limits of the reference ranges for all parameters and all centrifugation conditions (beta error)**. The results are displayed as outlined in the legend to table 4. Click here for file
